# Multi-modal representation of effector modality in frontal cortex during rule switching

**DOI:** 10.3389/fnhum.2015.00486

**Published:** 2015-09-22

**Authors:** Timothy L. Hodgson, Benjamin A. Parris, Abdelmalek Benattayallah, Ian R. Summers

**Affiliations:** ^1^School of Psychology, University of LincolnLincoln, UK; ^2^Department of Psychology, University of BournemouthBournemouth, UK; ^3^Exeter MR Research Centre, University of ExeterExeter, UK

**Keywords:** cognitive, executive, fMRI, manual, prefrontal, saccades

## Abstract

We report a functional magnetic resonance imaging (fMRI) study which investigated whether brain areas involved in updating task rules within the frontal lobe of the cerebral cortex show activity related to the modality of motor response used in the task. Participants performed a rule switching task using different effector modalities. In some blocks participants responded with left/right button presses, whilst in other blocks left/right saccades were required. The color of a Cue event instructed a left or right response based upon a rule, followed by a Feedback which indicated whether the rule was to stay the same or “Flip” on the next trial. The findings revealed variation in the locus of activity within the ventrolateral frontal cortex dependent upon effector modality. Other frontal areas showed no significant difference in activity between response epochs but changed their pattern of connectivity with posterior cortical areas dependent upon response. Multivariate analysis revealed that the pattern of activity evoked by Flip rule Feedbacks within an apparently* supra* modal frontal region (dorsolateral frontal cortex) discriminated between response epochs. The results are consistent with the existence of multi-modal representations of stimulus-response (SR) rules within the frontal cerebral cortex.

## Introduction

Performing many complex tasks involves the execution of actions based upon arbitrary mappings between stimulus and response. Previous patient and neuroimaging investigations have suggested that the lateral and medial frontal regions of the human cerebral cortex might be important in maintaining arbitrary stimulus-response (SR) rules and resolving response conflict in rule based tasks (Milner, 1963; Asaad et al., 1998; White and Wise, 1999; MacDonald et al., 2000; Passingham et al., 2000; Swainson et al., 2003; Boettinger and D’Esposito, 2005; Hampshire and Owen, 2006; Parris et al., 2007; Petrides, 2008; Wallis, 2008; Chouinard and Goodale, 2009). A key motivation of the current study was to investigate whether frontal regions anterior to the motor cortex show regional variation in activity dependent upon motor response modality (eye or hand) during a SR rule switching task. The alternative is that frontal regions encode information entirely supra-modally i.e., the *concept* of left or right, as opposed to the movement of a particular muscle group or effector organ to the left or right. If this were the case then activity should be independent of response modality.

A common assumption is that cognitive representations of task rules are maintained within the frontal cerebral cortex which modifies specific SR associations via top-down influences on sub-cortical and posterior cortical structures (Desimone and Duncan, 1995; Gehring and Knight, 2000; Miller and Cohen, 2001; Parris et al., 2007). This account predicts that given a task in which the same rule can be applied across more than one motor response modality frontal regions should show different patterns of connectivity with posterior cortical areas containing modality specific representations (e.g., for an eye movement or a hand movement). If cognitive representations of rules were truly supra-modal and distinct from representations of stimulus and response then they would not be able to exert top down control of action in this manner. Switching between different response modes given this type of representation would require an implausible state of neural “functional pleomorphism” in which individual neurons would need to actively modify the physical structure of their axonal network within a timeframe of seconds to select one type of response or another (Nachev et al., 2008).

At the same time a defining feature of human cognition is the ability to form abstract concepts and generalise across specific stimuli and contexts in a supra-modal manner (e.g., Deacon, 1997; Kosslyn et al., 2006). An important question therefore is how such representation is possible given the constraints of neural architecture described above. One possibility would be that even apparently supra-modal brain areas might possess modality specific tuning at a finer spatial scale such that sub regions or populations of neurons could selectively influence one or another mode of responding whilst activity across the entire population of neurons represents a general rule concept independent of specific responses to be executed. We term such a form of representation as “multi-modal” rather than truly supra-modal. If the spatial organization of such regions was consistent across participants at the local scale then functional magnetic resonance imaging (fMRI) should reveal their existence within the frontal cortex. However, if response specific sub-regions were idiosyncratically spatially distributed across participants, conventional univariate fMRI analysis averaging across individual participants may not be able to detect them. Another approach to delineate such an organization would be to examine the pattern of voxels activating in individual participants in different tasks. Woolgar et al. (2011) applied multi-voxel pattern analysis (MVPA) to fMRI data acquired whilst participants switched between different task rules and SR mappings linking four locations on a screen with index and middle finger key presses of the left and right hand. Whilst univariate analysis revealed no voxels with significant activity related to particular rules or SR associations, MVPA showed task rule related activity within the “multiple demands” cognitive network of the dorsal and ventral lateral frontal (Duncan and Owen, 2000).

In an earlier fMRI study, we examined brain activity during a color-response rule switching task (Parris et al., 2007). In this task a colored “Cue” presented at fixation instructed a left or right button press dependent upon an arbitrary rule (e.g., blue = left; yellow = right). The rule linking color and response can change dependent upon a “Feedback” signal which instructs participants to “Hold” or “Flip” the rule on the next trial. By jittering the timing of Cue, Response and Feedback events within a trial we were able to resolve BOLD responses to discrete events representing the response preparation and execution period and activity related to Flip/Hold rule Feedbacks. It was found that activity within dorsal and ventrolateral frontal areas, along with the medial frontal cortex was correlated with feedbacks that signaled the requirement to flip the rule on the next trial. Interestingly, a control condition revealed that lateral frontal regions activated equally strongly to infrequent/salient Feedback events which did not signal the need to update rules, whereas only the dorsal anterior cingulate cortex and basal ganglia responded selectively to the requirement to update SR mappings. It was concluded that lateral frontal regions are engaged in an evaluative process which determines whether current representations of rules need to be maintained or modified in the light of salient stimulus events, whereas medial frontal cortex implements updating of actual SR mappings.

We have also carried out studies of patients with neurological damage using the same SR rule switching task with either saccades or manual button press responses (Hodgson et al., 2007, 2013; Huddy et al., 2011). These suggest that some frontal regions mediating cognitive control operations might be segregated according to modality of response. Husain et al. (2003) tested a patient who had a highly localized lesion of the supplementary eye fields on both a manual and saccadic version of the color-response rule switching task. The patient was found to be selectively impaired in an oculomotor but not a manual version of the task such that they made increased errors following a rule change for saccades but not manual responses (Husain et al., 2003; Parton et al., 2007). Other patient work has shown that lesions to the ventrolateral frontal cortex bilaterally lead to significant increases in errors in a saccade version of the rule switching task (Hodgson et al., 2007).

Although a number of previous studies have investigated cognitive control mechanisms across sensory modalities (e.g., Roberts and Hall, 2008) few previous fMRI studies have compared activity between different motor modalities during cognitive tasks. These have revealed some evidence for the existence of sub-regions within the frontal cortex anterior to primary motor cortex organized by motor effector modality. For example, Leung and Cai (2007) compared two versions of a stop signal task for which participants could respond with either a left/right manual button press or a saccade. These authors found that Stop signal trials recruited the ventrolateral frontal cortex bilaterally under both conditions. However, saccade trials were associated with a more dorsal and anterior locus of activity whilst manual response trials recruited more ventral and posterior parts of the inferior frontal gyrus. Connolly et al. (2000) also compared stimulus directed and “anti” responding for saccades and manual pointing movements. They report that regions traditionally conceived of as oculomotor (e.g., FEF) are also activated during the programming of manual pointing responses. Whilst no regions were found to be selectively activated during anti-saccades relative to anti-pointing, some regions of the ventrolateral frontal cortex did show activity specific to manual pointing which was not observed during eye movement response blocks.

Although previous studies have examined different response modalities during cognitive tasks, no earlier work has compared activity between eye and hand movements during closely matched rule/task switching procedures. In our rule switching task described above (e.g., Parris et al., 2007), participants must intermittently update task rules in response to a Feedback signal. This affords participants the opportunity to reconfigure SR mappings in advance of the next response cue onset and at least some of the activity seen in response to “Flip” rule feedbacks may reflect this reconfiguration process. However, without comparison of activity across different response modes it is not possible to discriminate between activity related to reconfiguration of SR mappings and updating of supra-modal representations of rules. Differences in activity in response to “Flip” feedbacks between Eye and Hand movement versions of the task are likely to be related to reconfiguration of response specific task mappings in advance of the next trial. Moreover, such advanced reconfiguration of SR mappings might also be reflected in changes in the pattern of connectivity between frontal and posterior areas.

The study reported here used a color-response rule switching task closely based upon that used in our previous work (Hodgson et al., 2007; Parris et al., 2007). As in earlier studies the rule linking a colored cue and a left/right response could reverse several times during the course of the test, allowing us to examine event evoked activity during rule updating as well as response execution. Unlike our previous fMRI study each subject performed two versions of the task in alternating epochs within a single scanning run. In half the epochs participants responded with a left or right button press, whilst in the other epochs they executed saccades to the left or right. Data were analyzed at the group level to examine which regions activated to different task events in either a supra-modal or a response specific manner. Of particular interest was whether activity specific to “Flip” rule Feedback events was modulated by motor response (even though no motor response had to be executed). As it is possible that there might be no consistent pattern of spatial localization by response across participants we also explored individual participant data for evidence of response mode specific activity using multivoxel pattern analysis (Schrouff et al., 2013). Finally, we also examined changes in connectivity of frontal regions with response mode using psycho-physiological interaction (PPI) analysis.

## Materials and Methods

### Participants

Fifteen healthy right handed participants ranging in age from 18–36 years participated in the experiment. The study was approved by the School of Psychology Ethics Committee, University of Exeter and the Management Board of the Exeter Magnetic Resonance Research Centre, University of Exeter, UK. All participants gave written informed consent to participate in the study.

### Task and Procedure

Visual stimuli were presented on a back projection screen positioned at the foot end of the MRI scanner and viewed via a mirror mounted on the subject head coil. The screen subtended 16 degrees of visual arc to the subject lying prone in the scanner. Button press responses and manual reaction times were measured using two fiber optic button boxes held in the participants’ right and left hands. Eye movements were recorded using an Applied Science Labs 504 eye tracker with long range optics, mounted 1.5 m from the head end of the scanner bore, acquiring an eye image via a mirror fitted to the head coil. Eye position was sampled at 60 Hz and analyzed using custom written LabView software which allowed extraction of saccadic onset times, target-response latencies, movement direction and amplitude. Saccades were defined as periods for which eye velocity exceeded 30°/s for three consecutive samples and exceeded a displacement criterion of one degree of visual arc from the eye starting position.

**Figure 1:**
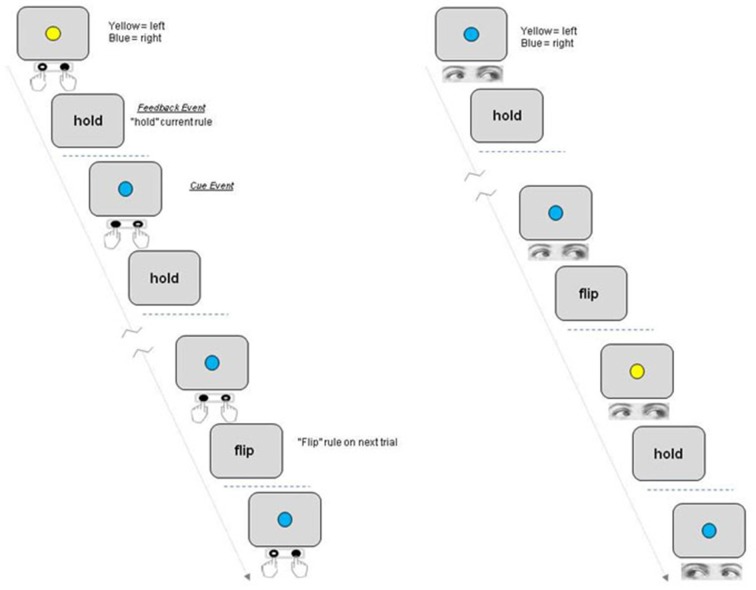
**Schematic of rule switching task.** Participants switch between two rules linking a centrally presented colored Cue with a motor response to the left or right. The participant either holds or reverses the rule linking cue and response on the next trial based on the Feedback event following response execution (“Hold”/“Flip”). Each participant completed four blocks, two blocks each for which either Manual press button responses (“Hand epochs”) or saccades were made to the left or right (“Eye epochs”).

A schematic of the task is shown in Figure 1. Each trial in the task began with the presentation of a blue or yellow colored circle (the response contingent* Cue event*). Subjects made a left or right behavioral response dependent upon the color of the cue and the current rule linking color with direction. For “Eye” (saccade) blocks participants were instructed to fixate one of two peripheral response locations (marked by two onscreen response boxes located approximately five degrees to the left or right of the central location and displayed during Eye epochs only). When the cue stimulus was extinguished they then had to make a return saccade to fixate the central location. The word “hold” or “flip” was then presented at fixation to indicate to the participant whether to hold or reverse the current rule on the following trial (*Feedback event*). In order to resolve the fMRI response to these discrete events within a trial i.e., Cue and Feedback, the period between each event and trial was varied randomly between 2200, 4400 and 10200 ms such that inter-event intervals did not correspond to multiples of the TR period. Varying the period between individual events in this manner within as well as between trials ensures that discrete events can be resolved within a trial (Parris et al., 2007).

Each block consisted of 21 trials and each subject completed four blocks in one continuous scanning run, with two blocks requiring responses to be made with a manual press button response (*Hand* blocks) and two blocks required a saccadic response to be made to a response box located to the left or right of the central location (*Eye* blocks). Eye and hand blocks were presented alternately with half the subjects completing the task in the order *Eye-Hand-Eye-Hand* and the other group of subjects in the order *Hand-Eye-Hand-Eye*. Each task block was preceded by an instruction screen which told subjects to either “Respond with Eyes” or “Respond with Hands” as well as showing the rule to be used on the first trial (e.g., blue = respond left; yellow = respond right) which was displayed for 10 s, followed by a variable delay period before presentation of the first colored cue stimulus. Participants completed one practice block of each version of the task outside the scanner just prior to the scanning session.

### fMRI Data Acquisition

Scanning was performed on a 1.5 T Philips Gyroscan magnet at the Peninsula MR Research centre, University of Exeter, UK. A T2^*^-weighted echoplanar sequence was used (TR = 3000 ms, TE = 50 ms, flip angle 90°, 32 transverse slices, 3.6 × 3.6 × 4 mm, ascending acquisition). Four hundred and sixty five image volumes were acquired in one continuous scanning run. An additional five “dummy” scans were performed at the start of each block prior to the start of the stimulus sequence.

### fMRI analysis

#### Univariate Analysis

Data were analyzed using SPM8 software. The images were realigned, unwarped to remove variance caused by movement-by-field-inhomogeneity interactions, normalized to a standard EPI template, and smoothed with a Gaussian kernel of 6 mm full-width at half maximum. Inter-event periods were selected such that the response Cue and Feedback event sequences were temporally de-correlated with each other and the TR period (3000 ms TR relative to 2200, 4400 and 10200 ms inter-event period see *task and procedure* above), This design allowed unique regressors to be derived for each event type by convolving the event onset times with a canonical hemodynamic response function. The eight different event types modeled at the individual subject level were:

*Response Cue event onsets: Hold rule-Hand; Flip rule-Hand; Hold rule-Eye; Flip rule-Eye; Feedback event onsets: Hold rule-Hand; Flip rule-Hand; Hold rule-Eye; Flip rule-Eye*.

Response error events were also modeled for participants who made such errors. Separate regressors were included in the first level model corresponding to the timing of *Hand response errors* and* Eye Response errors*. Trials on which errors occurred were not represented in the regressors corresponding to the main event and trial types listed above.

A second level (random effects) factorial statistical model was applied to the one sample *t*-tests derived from each of the regressors described above for each individual subject’s data. A *three-way analysis* of variance included three factors with two levels each: event type (Cue vs. Feedback); Trial type (Hold vs. Flip rule) and Response type (Hand vs. Eye). All factors and levels were assumed to be non-independent (repeated measures) and the statistics and degrees of freedom used for inference were calculated accordingly. Two approaches were taken in the comparison of activation by response. Direct statistical contrasts between conditions of interest were first carried out (two sample *t*-tests testing the main effects of Response and *F*-tests for interaction effects of Trial type with Response). In addition, an exclusive masking approach was also taken to explore response mode specific activity in which activity for one response type was masked with the equivalent contrast for the other response using a very low statistical threshold (*p* < 0.05 uncorrected) to derive the exclusive mask (e.g., Vilberg and Rugg, 2008). We reasoned that this exclusive masking approach would be more likely to identify regions which show significant activation differences in some participants but which do not consistently generalise across participants, whereas direct statistical contrasts would only reveal areas which showed highly consistent response specific activations across participants.

For key comparisons of interest, we also applied a number of additional inclusive and exclusive masking and region of interest (ROI) analyses derived from other relevant contrasts (e.g., Flip vs. Hold Feedback event contrast masked by the main effect of Cue event activity for Eye epochs) or *a priori* hypotheses arising from previous fMRI studies (e.g., Leung and Cai, 2007; Parris et al., 2007). In the case of all contrasts utilizing a mask or ROI a Small Volume Correction (SVC) was applied to determine which voxels survived correction for multiple statistical comparisons. Anatomical ROIs were generated using the Wake Forest University (WFU) Pickatlas toolbox (Maldjian et al., 2003).

The conjunction analyses across Eye and Hand epochs tested the conjunction null hypothesis (see Friston et al., 2005; Nichols et al., 2005 for discussion of global vs. conjunction null hypothesis testing).

In all cases only those activations which passed a significance criterion of *p* < 0.05 Family Wise Error (FWE) corrected for multiple comparisons are reported below and the results of all other statistical contrasts carried out at the individual or group level revealed no significantly activated voxels based upon this criteria. All coordinates given in tables are Montreal Neurological Institute (MNI) coordinates. Anatomical labels and estimated Brodmann area numbers were generated by converting MNI coordinates to Talaraich and interrogation of the Talaraich Daemon database tool (Lancaster et al., 2000).

#### Psycho-Physiological Interaction Analysis

In order to assess the hypothesised biasing influence of frontal areas on posterior brain regions during updating of SR rules, a PPI analysis was carried out (Friston et al., 1997). PPI analyses assess whether the correlation in activity between two distant brain areas differs dependent upon psychological context—in other words, whether there is an interaction between a psychological variable and the functional coupling between two brain areas. In this case the “psychological” variable was whether a saccade or a press button response had to be made during a given epoch and the physiological variable was the BOLD signal in a given spherical volume of interest (VOI) or “seed” region. Two seed VOIs in the frontal cortex were used in the analysis reported below based upon the first level conjunction analysis for regions activated equally by Flip rule Feedbacks during both hand and eye epochs.

#### Multi-Voxel Pattern Analysis

Multivariate analysis of the data was carried out to investigate whether differences between response epochs were apparent in the pattern of activity across voxels rather than within individual clusters of voxels. The Pattern Recognition Neuroimaging Toolbox (PRoNTo; Schrouff et al., 2013) was used to implement a binary classifier based upon the support vector machine approach (Cristianini and Shawe-Taylor, 2000) using realigned, unwarped, normalized, unsmoothed data from each participant (use of spatially smoothed data not being recommended with MVPA/PRoNTo). In simple terms, the classifier algorithm works out the best array of weightings to apply to voxels within the specified mask in order to discriminate between two experimental variables (In the present case whether the motor response to be executed in response to the next Cue event was to be an Eye or Hand movement). Activity elicited by Flip feedback events was analyzed in this way to determine whether the overall pattern of activating voxels differed systematically between the two response modality conditions. In order to avoid the classifier algorithm “over fitting” the data PRoNTO utilizes a procedure in which the classifier is tested for discrimination accuracy on a subset of the total scan run which was not used during the “learning” phase, a so-called “leave-one-block-out” approach to cross validation within a single dataset.

## Results

### Behavioral Data

None of the participants made any errors in which the wrong type of response was initiated in a given epoch (i.e., an eye movement when a manual response was required and vice versa). Directional errors in eye and hand responding were also very rare. A total of five such errors were made in Eye response blocks across all subjects and six manual response errors equating to <4% errors overall. For saccadic, but not manual responding, “corrected” errors were observed on some trials in which a saccade was initially directed in the wrong direction but the movement was quickly corrected such that the eye was directed to the correct response box. A total of only 10 such corrected errors were observed across all participants. Means and standard errors of response times across participants for eye and hand blocks were 865 ± 41 ms and 774 ± 46 ms respectively. A two-way ANOVA with response type and trial type (Hold/Flip rule), revealed no significant effect of response type, trial type or interaction between response and trial type on reaction times.

### fMRI analysis

#### Main Effect of Trial Type (Hold vs. Flip Rule)

No voxels were significantly activated for the comparison of Trial Type (Hold > Flip or Flip > Hold) for Cue events. The comparison of Flip compared to Hold rule trial Feedback events highlighted superior bilateral parietal cortex, the anterior cingulate, left middle frontal gyrus, left inferior frontal gyrus and bi-lateral cerebellum (Table 1). The reverse contrast (Hold > Flip) revealed no significantly activated voxels.

**Table 1:** **Coordinates of peak activations clusters for the contrast of “Flip” vs. “Hold” trial Feedback events**.

Location			Cluster size	Anatomical location	*Z* score	*p* value (FWE corrected)
−30	−62	46	305	Superior parietal lobe (BA7)	6.67	0.0001
8	−76	40	109	Medial parietal lobe, precuneus (BA7)	6.30	0.0001
−30	22	−6	62	Inferior frontal gyrus (BA47)	5.61	0.001
−51	−44	40	84	Inferior parietal lobe (BA40)	6.04	0.001
−38	3	56	57	Middle frontal gyrus (BA6)	5.25	0.005
−36	−60	−32	57	Left cerebellum	4.98	0.019
36	−54	−38	24	Right cerebellum	5.05	0.014
−4	20	46	16	Anterior cingulate gyrus (BA32)	5.03	0.015
−46	34	24	14	Middle frontal gyrus (BA9)	4.80	0.04

*Data collapsed across Eye and Manual response epochs. MNI coordinates given for activations surviving FWE correction for multiple comparison at p < 0.05*.

#### Main Effect of Response Type (Hand vs. Eye)

Areas which showed more or less activity during the two response mode epochs (Eye/Hand) were compared via both direct statistical contrasts and the exclusive masking technique for both Cue and Feedback elicited event activations.

**Figure 2:**
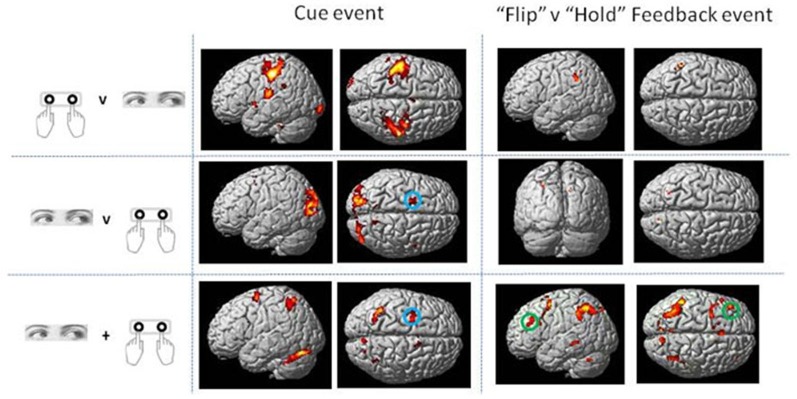
**Rendered surface activation views of event locked activity for main effect of Cue and contrast of Flip minus Hold Feedback events which was either exclusive to Hand or Eye response periods or overlapping between the two response modes (conjunction analysis).** The exclusive masking approach was used to compare Eye and Hand activations. See Table 2 for activation coordinates, anatomical labels and Brodmann Area classification. Green circle shows dorsolateral frontal cortex locus used as the basis of the Psycho-physiological interaction analysis (PPI) and MVPA (BA 9/46). Blue circle indicates region activated by Cue events corresponding to the location of human frontal eye fields (FEF; BA6). Activations are shown thresholded at *p* < 0.05 FWE corrected.

Direct statistical contrasts for Feedback and Cue evoked activity (independent of Trial type) revealed no differences between Eye and Hand blocks. Cue locked activity in Eye blocks masked by Hand blocks showed activity in the left frontal eye fields (FEF), medial parietal and occipital cortex along with the Middle temporal and parahippocampal gyrus (Table 2; Figure 2). The reverse comparison of Cue related activity in Hand epochs exclusively masked by Eye epochs highlighted the bilateral post-central gyrus, cerebellum and middle occipital gyrus (Table 2; Figure 2). For Feedback evoked activations exclusive masking analysis showed Hand epoch activity exclusive to the right post-central gyrus and left inferior frontal gyrus. No activity evoked by Feedback events was found to be exclusive to Eye blocks.

**Table 2:** **Cue event activations which were greater for Eye vs. Hand or Hand vs. Eye response epochs**.

Location			Cluster size	Anatomical label	*Z* score	*p*-value (FWE corrected)
**Eye > Hand**
−24	−4	54	84	Left middle frontal gyrus BA6	6.56	0.0000
−20	−4	62		Left middle frontal gyrus BA6	5.36	0.0032
−18	6	64		Superior frontal gyrus BA6	4.83	0.0354
−20	−82	42	977	Precuneus BA7	6.54	0.0000
−26	−90	20		Cuneus BA19	6.45	0.0000
−14	−92	36		Cuneus BA19	6.39	0.0000
−24	−58	−6	40	Parahippocampal gyrus BA19	6.28	0.0000
38	−82	18	327	Middle temporal gyrus BA19	6.15	0.0000
18	−90	40		Precuneus BA7	5.93	0.0002
30	−86	30		Cuneus BA19	5.64	0.0008
30	−58	−8	27	Parahippocampal gyrus BA19	6.02	0.0001
12	−56	54	40	Precuneus BA7	5.48	0.0017
**Hand > Eye**
−50	−26	50	192	Postcentral gyrus BA2	5.99	0.0001
−50	−18	46		Postcentral gyrus BA3	5.91	0.0001
20	−104	−8	59	Cuneus BA18	5.83	0.0002
34	−98	−6		Middle occipital gyrus BA18	5.18	0.0059
28	−102	−2		”	5.15	0.0065
−50	−10	14	67	Precentral gyrus BA14	5.39	0.0021
−52	−18	16		Postcentral gyrus BA43	5.12	0.0078
42	−34	66	73	Postcentral gyrus BA3	5.37	0.0023
52	−30	60		Postcentral gyrus BA2	5.07	0.0097
52	−22	10	53	Transverse temporal gyrus BA41	5.22	0.0047

#### Interactions Between Trial Type and Response Type

A key motivating question for the study was whether the location of regions showing an enhanced response to Flip relative to Hold rule Feedback events varied dependent upon response type. Of particular interest is whether the increase in activity observed in some areas during Flip relative to Hold Feedback events was modulated by motor response (even though no motor response was actually executed contingent upon this signal to implement a new rule on the next trial). Activations of this type would be indicative of rule updating and cognitive control processes which were specific to particular modalities of response.

**Figure 3:**
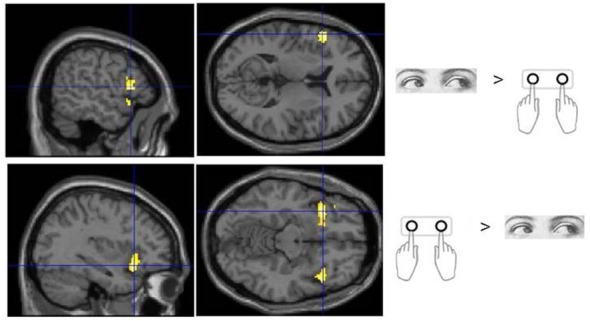
**Sub regions of the ventrolateral frontal cortex which were found to show activity which varied with response modality during processing of Flip rule feedback events**.

No voxels were found whose activity depended upon the interaction effect between Response and Trial type, even when an inclusive mask corresponding to the main effect of Flip minus Hold rule Feedbacks and an appropriate SVC was applied. Using the exclusive masking approach, in which the contrast of Flip minus Hold trial feedback was exclusively masked with regions showing Eye/Hand response (i.e., *Cue* event) locked activity, a number of regions were found to be significantly activated suggesting that these response execution related regions were also activate during rule updating (Figure 2; Table 3A). Previous research had identified areas of the ventrolateral frontal cortex as showing response specific activity during cognitive tasks including stop signal and anti saccades/anti pointing tasks. In order to test whether a similar organization could be observed during rule switching, an ROI encompassing the bilateral ventrolateral frontal cortex (combination of BA45, BA47, BA44 as defined within the WFU Pickatlas toolbox) and associated SVC was applied to the data. This revealed that Hand response epochs were associated with a more ventral and posterior locus of activity, whereas for the reverse comparison of Eye epochs exclusively masked with Hand epochs, a more dorsal and anterior locus confined to the left hemisphere was highlighted (Table 3B; Figure 3).

**Table 3:** **(A)** Coordinates of peak activations for Flip vs. Hold rule Feedbacks within regions which showed significant Cue response related activity specific to either Eye or Hand epochs (*p* < 0.05, whole brain FWE corrected). **(B)** Coordinates of activation within the ventrolateral frontal cortex which showed response mode exclusive activity during processing of rule change feedback events. **(C)** Psychophysiological interaction (PPI) analysis for lateral frontal cortex seed region connectivity with posterior cortical areas during processing of rule change feedback events in Hand versus Eye blocks.

Location			Cluster size	Anatomical label	*Z* score	*p*-value (FWE corrected)
**(A) Eye > Hand**
−50	−46	36	45	Inferior parietal lobule BA 40	5.39	0.002
−50	−42	46	23	”	4.82	0.03
−46	−54	50	3	”	4.83	0.028
−50	−44	50	1	”	4.73	0.043
−52	−40	−4	1	Middle temporal gyrus BA22	4.92	0.019
20	−70	−26	2	Right cerebellum, Declive	4.78	0.035
**Hand > Eye**
−22	−62	46	12	Superior parietal lobule BA7	5.87	0.000
−30	−66	52	1	”	4.70	0.048
12	−74	42	7	Precuneus BA7	5.06	0.01

**(B) Eye > Hand**
−58	18	12	93	Inferior frontal gyrus BA45	4.44	0.014
−50	20	10		”	4.40	0.016
**Hand > Eye**
−34	20	−12	315	Inferior frontal gyrus BA47	5.04	0.001
46	18	−12	247	”	4.74	0.022

**(C) Eye > Hand**
22	−98	−10	72	Lateral occipital gyrus BA18	4.56	0.039
**Hand > Eye**
40	−72	−31	248	Angular gyrus BA39	4.47	0.002

#### Conjunction Analysis (Common Activations Between Eye and Hand Epochs)

Areas which showed completely overlapping event elicited activity during the two response modes (Eye/Hand) were examined for each relevant event type contrast using the conjunction null hypothesis test option in SPM8.

This analysis for Cue locked activity alone revealed predominantly left superior parietal cortex activity, bilateral cerebellar and dorsolateral frontal (BA6) activity in the region of the FEF (Mort et al., 2003; Figure 2).

The conjunction analysis for the contrast between Flip vs. Hold Feedback events (i.e., rule updating vs. non-rule updating events) for Hand and Eye epochs showed activity in the medial frontal cortex including activation peaks within the dorsal anterior cingulate gyrus, superior frontal gyrus, left inferior frontal cortex/frontal operculum (BA47), bilateral inferior parietal cortex and left dorsolateral frontal cortex (BA9/BA46; Figure 2).

#### Psycho-Physiological Interaction Analysis

In order to assess the hypothesised biasing influence of prefrontal cortical areas on other brain regions during updating of rules and how this might vary with response modality, a PPI analysis was carried out (Friston et al., 1997). Two analyses were carried out for each participant using spherical VOIs as seed regions (radius 15 mm) centered around the peak activation coordinates within the left dorsolateral frontal cortex (BA9: −46, 34, 26 MNI space) and dorsal anterior cingulate gyrus (BA32: −4, 20, 46) as highlighted by the conjunction analysis above (Figure 2). An ROI and corresponding SVC, comprising the entire parietal and occipital lobes were applied to the results combined with a FWE correction for multiple comparisons.

**Figure 4:**
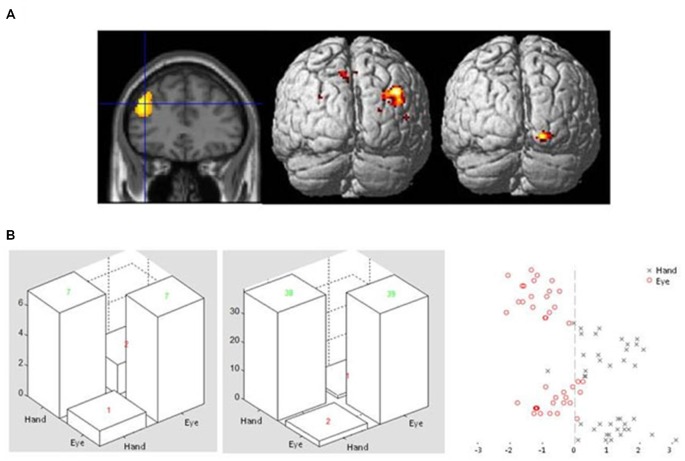
**(A)** Sectional view of activity in dorsolateral frontal cortex ROI during Flip rule feedback events (left panel) used as a seed region in a PPI analysis, alongside rendered view of posterior cortical areas showing enhanced covariance with the seed region in Hand (middle) or Eye (right) response epochs. **(B)** Examples of confusion matrices and classifier function plots illustrating typical discrimination performance of a voxel pattern analysis classifier discriminating between Flip feedback event related activity in either Eye or Hand response epochs in the same left dorsolateral frontal ROI.

This analysis revealed a large cluster of voxels in the right posterior parietal cortex which showed enhanced connectivity with the lateral frontal seed region during Hand compared to Eye epochs. For the reverse comparison, a cluster of voxels in the lateral occipital cortex showed significantly enhanced covariance of activity during eye relative to hand epochs (Figure 4A; Table 3C). No significantly activated voxels were apparent from the equivalent analysis using the anterior cingulate gyrus as the seed region.

#### Multi-Voxel Pattern Analysis

Finally, we applied pattern analysis/classifier algorithms to the data set to assess whether the overall pattern of activity across voxels was different for feedback events which occurred during Eye or Hand epochs (even though the occurrence of a Feedback event was not a direct signal for execution of a motor response). In particular, we wanted to apply this technique to regions which the univariate analysis indicated were supra-modal in nature (such that no voxels showed significant variation in activity dependent upon response epoch) but which nevertheless showed evidence for changes in connectivity with posterior regions during rule updating.

A binary support vector machine classifier was applied to Flip feedback event related activity in voxels extracted from the same left dorsolateral frontal ROI used as a seed region in the PPI analysis (see above). Using this approach it was found that the machine classifier was able to correctly identify whether activity evoked by Feedback events occurred in Hand or Eye epochs. This was found to be the case for all participants tested, with an overall mean accuracy across subjects of 85% (ranging from 75–98 between participants) for Hand and 84% (62–98) for Eye response epochs (Figure 4B).

## Discussion

A key motivation of this study was to examine whether regions of the frontal cerebral cortex previously shown to be active in a SR rule switching task might show regional variation in activation patterns dependent upon effector modality (saccades vs. manual button pressing). As would be expected, activity dependent upon the type of response (Eye/Hand) to be executed time-locked to response Cue events was found within the inferior parietal cortex, lateral occipital cortex, post-central gyrus and FEF (Table 2). Interestingly however, some of these eye/hand dependent regions were also active during processing of rule Feedback events, for which a motor response was not required but cognitive updating of SR rules was (Table 3). Consistent with earlier fMRI studies, eye/hand specific sub regions were apparent within the ventrolateral frontal cortex (Table 3B). Further analysis also suggested that even apparently supra-modal regions within the lateral frontal cortex changed their pattern of connectivity with posterior areas during rule updating (PPI analysis) and showed variation in the overall pattern of voxel activity dependent upon response modality (PRoNTo/MVPA analysis).

Our results support previous findings which suggest that sub-regions of the ventrolateral frontal cortex support cognitive operations for particular motor domains specifically the inferior frontal gyrus pars triangularis and orbitalis. A more dorsal and anterior locus of activity was found for saccade responses, whilst manual response trials recruited ventral and posterior parts of the inferior frontal gyrus bilaterally. The inferior frontal cortex has been suggested as an important substrate of cognitive inhibitory control across different modalities (Aron et al., 2014) as well as forming part of a general multiple cognitive demands fronto-parietal network (Duncan and Owen, 2000). In a previous fMRI study using a manual response version of the rule switching task we found the region to be active even in a control task where “flip” feedbacks did not require a rule reversal (Parris et al., 2007). From this it was concluded that the role of this region was related to processing and evaluating salient stimulus events rather than updating SR rules or implementing cognitive inhibitory control (see also Aron et al., 2014; Erika-Florence et al., 2014). However, the current results suggest that this region *does* have an underlying motoric organization and function.

The conjunction analysis highlighted the dorsolateral region of the frontal cortex as being equally activated during rule updating for both eye and hand versions of the task. A suggested function of the dorsolateral frontal cortex is that it applies appropriate biasing input onto posterior structures to ensure that behavior adaptively conforms to current goals (Desimone and Duncan, 1995; MacDonald et al., 2000; Miller and Cohen, 2001). To assess evidence for such changes in connectivity between epochs a PPI analysis was carried out. This revealed clusters of voxels within the occipital and parietal cortex whose activity covariance with the dorsolateral frontal cortex was significantly modulated by response type (Table 3; Figure 4). This result prompts the question as to how truly supra-modal neural representations could influence posterior cortical areas in a modality specific manner without modifying their pattern of anterior to posterior synaptic connections over a very short time scale (Nachev et al., 2008).

One way of answering this question is offered by recent work which has suggested that standard univariate fMRI analysis lacks the resolution required to detect neuronal organization by rule SR associations, whereas multivariate techniques such as MVPA are better able to discriminate between different task rule related activity (e.g., Woolgar et al., 2011). Consistent with this, an MVPA analysis within the same left lateral frontal ROI used in the PPI analysis was able to discriminate between Hand and Eye response blocks with a high degree of accuracy. This is consistent with the existence of distributed multi modal representations of motor response type even within regions which at the voxel cluster level activate equally during Eye and Hand movements. It should be noted however that other authors have argued that the MVPA comparisons across task conditions may be susceptible to unknown confounds which vary across participants, such as task difficulty and response times (Todd et al., 2013). Although we believe the overall level of task/rule complexity is closely matched between the two conditions we acknowledge that MVPA discrimination based on factors other than response modality related tuning cannot be entirely excluded.

The conjunction analysis for *Cue* locked activity revealed a number of areas which activated equally during response programming and execution irrespective of the modality of the response to be executed. These included areas of the bilateral superior parietal cortex, the ventrolateral occipital cortex and lateral cerebellum (Figure 2). It is likely that these regions represent aspects of responding to the Cue stimulus which did not relate directly to the execution of a response. For example, processing the color of the cue and selection of the associated spatial concept i.e., left or right, independent of response mode. It is interesting that activity within the region assumed to be the location of the FEF (Mort et al., 2003) was observed in both hand and eye epochs (Figure 2). None of our participants made any errors in which the wrong* type* (rather than the wrong *direction*) of response was executed (e.g., an overt saccade rather than a button press in manual response epochs). Other neuroimaging studies which have studied cognitive task execution with hand and eye movements have also reported FEF activity during manual as well as saccade response blocks (Connolly et al., 2000). One interpretation of this finding is that oculomotor centers play a role in orienting of spatial attention and manual response programming and that the oculomotor system is active during orienting of attention irrespective of the overt response mode required by the task (Rizzolatti et al., 1994).

## Summary and Conclusions

During a SR rule switching task the lateral frontal cortex was found to show regional variation in activity dependent upon the type of motor response to be executed (Hand or Eye), even during events that signaled a change in rule rather than being associated with an actual motor response to be executed. Closer examination of fMRI data shows that even within frontal areas which univariate analysis suggested were equally activated during rule switching with manual or saccade responses, differences were apparent between Hand and Eye response epochs in terms of their connectivity with posterior areas as well as the overall pattern of voxels activated within the region. We suggest that higher order representations of task rules are underpinned by neural representations which represent effector modality. Such a distributed form of representation is best termed multi modal rather than supra-modal and may not always be apparent when standard univariate statistical analysis techniques are applied to fMRI data.

## Conflict of Interest Statement

The authors declare that the research was conducted in the absence of any commercial or financial relationships that could be construed as a potential conflict of interest.
